# Spectrum, Pattern, and Clinical Outcomes of Adult Emergency Department Admissions in Selected Hospitals of Western Ethiopia: A Hospital-Based Prospective Study

**DOI:** 10.1155/2019/8374017

**Published:** 2019-08-06

**Authors:** Ashenafi Habte Woyessa, Birhanu Yadecha Dibaba, Getahun Fetensa Hirko, Thanasekaran Palanichamy

**Affiliations:** ^1^Wollega University, Institute of Health Science, School of Nursing and Midwifery, Department of Emergency and Critical Care Nursing, Nekemte, Ethiopia; ^2^Wollega University, Institute of Health Science, School of Nursing and Midwifery, Department of Nursing, Nekemte, Ethiopia; ^3^Wollega University, Institute of Health Science, School of Nursing and Midwifery, Department of Psychiatry Nursing, Nekemte, Ethiopia

## Abstract

**Background:**

There has been a steady rise in the absolute number of emergency room admissions over the last few decades. The healthcare delivery system of a country is required to be adjusted to patterns of morbidity and mortality to mitigate the minimized prolonged ill health consequences and premature death of adults. The spectrum, patterns, morbidity, and mortality of health and health-related emergency conditions for which patients visit hospitals often reflect the magnitude of different health problems in a society. The objective of this study was therefore to assess the spectrum, pattern, characteristics, and clinical outcomes of emergency department admissions among adult people who visited EDs of the selected hospitals in western Ethiopia.

**Methodology:**

Hospital-based prospective cross-sectional study design was utilized. To select hospitals to be included in the study, the area sampling technique was used. Five administrative zones in west Oromia were selected as geographical clusters. Then, four hospitals were randomly selected from each zone. Finally, the consecutive sampling technique was utilized to recruit the study participants.

**Results:**

The mean age of the patients admitted to emergency departments (EDs) of the selected hospitals was 34.98 years. The male-to-female ratio of the respondents was nearly equal (1 : 1.04). While one-fourth (20.4%) of the patients arrived by ambulances (without identifying reason), 23.6% of them visited the emergency department as they had no other place to go. Medical emergencies (45.4%) were the leading types of emergencies followed by traumatic emergencies (27.3%). Respiratory distress (12.43%), extremity fractures (9.61%), and hypertensive disorders (8.6%) were among the top leading causes of adult ED admissions. Vital signs were deranged in about 59.4% of the cases. The most common type of immediately life-threatening problems identified on arrival was impairment of breathing (37%), followed by circulatory compromises (30%). Emergency department admission patterns were variable with peak admissions in the month of February and the lowest in November. The vast majority (90.9%) of emergency patients survived. While 8.5% of patients died of the various types of emergency conditions, the final clinical outcome was not identified in 1.5% of the patients.

**Conclusion:**

This study has showed mixed cases with varied patterns and outcomes of adult emergency department admissions. As overall there is a need to be alert during specific seasons, actions must be taken to improve the readiness of existing emergency room services. Furthermore, it is worthwhile to invest further on standardizing and organizing prehospital services at the community level.

## 1. Introduction

The health of adults in sub-Saharan Africa is becoming increasingly important priority in global health policy. There has been constant increase in the absolute number of emergency admissions for defined populations over the last three decades. Adult mortality, death between the ages of 15 and 60, is 4 to 40 times higher in sub-Saharan Africa than in developed countries [1, 2].

The causes of morbidity and/or mortality in sub-Saharan Africa regions are predicted to undergo a significant shift towards endemic noncommunicable diseases. A gradual change in lifestyle and improvement of socioeconomic status of the third world countries are causing substantial changes on the previously recognized public health problems. Public violence including violence to women is also becoming important causes of emergency department visits. Moreover, poorly organized emergency medical service system where prehospital services are totally lacking in several places calls for considering modifications of the adapted healthcare delivery system. In other words, the healthcare delivery system of a country must be adjusted to patterns of morbidity and mortality to mitigate and minimize consequences of prolonged ill health and premature death of adults [3, 4].

Information related to the spectrum and patterns of emergency conditions for which clients visit the emergency departments and their clinical outcomes often indicates the actual magnitude of different health problems in a community. Furthermore, such information are essential in healthcare planning and provision of essential health services including key resources like equipment, hospital space, and other logistics [5–9].

However, very little or probably nothing has been known about the spectrum, pattern, characteristics, and clinical outcomes of local-community emergency problems for which they visit emergency rooms in Ethiopia. The objective of this study was therefore to assess the spectrum, pattern, characteristics, and clinical outcomes of emergency department admissions among adult people who visited EDs of the selected hospitals which ultimately contribute to works done to reduce the ever-growing mortality and complications secondary to emergencies.

## 2. Materials and Methods

### 2.1. Study Setting

This study was conducted in three zones of Wollega, located in the western part of Oromia regional state, western Ethiopia. Four zonal hospitals with a similar level of services were randomly selected from these zones: Nekemte Referral Hospital from East Wollega Zone, Gimbi and Nejo Hospitals from West Wollega Zone, and Shambu Hospital from Horo Guduru Wollega Zone. The study was carried out from February, 2017 to June 2017 among adult patients who have visited the selected hospitals for all forms of emergency conditions.

#### 2.1.1. Methods

Hospital-based prospective cross-sectional study was employed to assess the pattern, spectrum, characteristics, and clinical outcomes of adult emergency department admissions in the selected hospitals.

#### 2.1.2. Sample Size Determination and Sampling Procedure

Since the objective of the study was to determine the spectrum, patterns, and clinical outcomes of adult emergency department visits during a specified study period, no specific sampling size determination was employed. The area sampling technique was used to select the zones in which the hospitals are found. Five administrative zones, namely, Buno, Kelem Wollega, West Wollega, East Wollega, and Horo Guduru Wollega zones were selected as geographical clusters from the western Oromia region. Out of them, three zones (West Wollega, East Wollega, and Horo Guduru Wollega) were randomly selected. Then, the four mentioned hospitals were randomly included in the study. Finally, all adult patients with all forms of emergency problems who have visited and were treated in emergency departments of the respective hospitals during the study period were consecutively included.

#### 2.1.3. Inclusion Criteria

All adult patients who have visited the ED of the selected hospitals for all emergency conditions during the study period were included.

#### 2.1.4. Exclusion Criteria

All patients of less than 18 years old and who were critical and unconscious patients with no attendants were excluded.

#### 2.1.5. Research Tool and Data Collection Techniques

Data were collected using comprehensively organized and pretested interviewer-administered questionnaires. The tool was adopted from up-to-date literature [2, 3, 5, 6, 10, 11]. Necessary modifications were made to the tool so that it was specific, reliable, and valid enough to answer the research questions and meet the study objectives. The questionnaire was composed of four parts (sociodemographic characteristics of the patients, baseline clinical information, spectrum, pattern of visits, and Emergency Department visit outcomes). Eight data collectors (two for each hospital) were recruited. In order to familiarize them with collection of the required data and upholding the confidentiality, training was given by investigators.

#### 2.1.6. Data Quality Control

Initial tool prepared in English was translated to local language (Afan Oromo) and translated back to English by respective language experts. Before conducting the main study, pretest was carried out in one nonselected hospital. Based on the findings of the pretest, necessary modifications were made to the tool. In addition, investigators have closely supervised the data collection process and necessary corrections were considered on all data collection sites. Moreover, the collected data were checked again for completeness before data entry. All incomplete data found were discarded. The cleaning process was performed by running simple frequency after data entry for its consistency. Further check has been done for inconsistency by referring the hard copy of the questionnaires.

### 2.2. Data Processing and Analysis

After data collection, the already coded questionnaires were checked for completeness. Data were initially entered into EpiData version 3.2 by data clerks after several steps of check for completeness and accuracy. Then, the entered data were exported to SPSS program version 20 for data analysis. Descriptive statistics was generated for spectrum, characteristics, patterns, and clinical outcomes emergency department admissions. Finally, the results of the study were presented using text, tables, charts, and graphs.

## 3. Result

A total of 952 adult patients presented to the emergency departments of the selected four hospitals in western Ethiopia and who met the inclusion criteria were involved in the study. As incomplete questionnaires were discarded, data obtained from 889 respondents were analyzed, making the response rate 93.3%. The key findings related to sociodemographic characteristics, baseline clinical information, spectrum and forms of ED admissions, leading causes of adult ED admission, and final outcome of adult ED admissions are presented in the subsequent sections.

### 3.1. Sociodemographic Characteristics of the Patients

The mean (+SD) age of the patients admitted to adult emergency department of the hospitals was 34.98 (+15) years. The male to female ratio of adult emergency department admissions was nearly equal (1 : 1. 04) (Table 1).

### 3.2. Baseline Clinical Characteristics of the Patients

This study has identified the baseline clinical characteristics of patients and reasons of emergency room visit. Out of total respondents, more than half (51.9%) of the patients were presented to the EDs as self-referral, whereas the remaining patients were referred to the hospitals from other health facilities. It was their first visit to the ED of the selected hospitals for over three-fourth (76.9%) of the patients. While levels of consciousness in about 10% of emergency patients were not determined, 127 (14.3%) of patients arrived unconscious. Vital signs were deranged in about 59.4% of the cases (Table 2).

### 3.3. Reasons of Emergency Department Visits

The study also explored respondents' relevant reasons of emergency room visits. While only one-fourth (20.4%) of them arrived by ambulance or other emergency vehicles, only 40 (4.5%) of them visited the ED because they believe that they get most of care at the emergency room (Table 3).

### 3.4. Patterns and Spectrum of Emergency Department Admission

**Figure 1:**
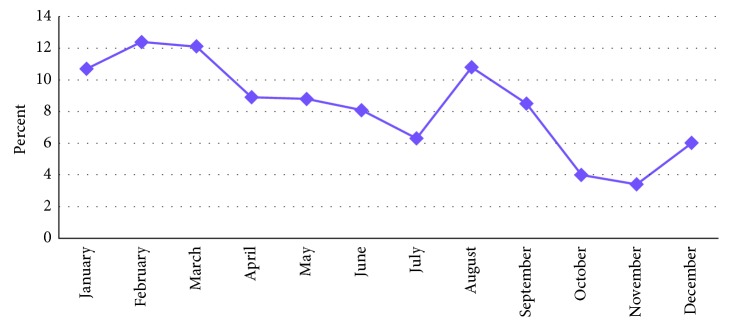
Patterns of adult emergency department admissions in Nekemte, western Ethiopia, 2017.

In this study, the number of adult ED admissions per month was variable with peak admissions in the month of February and the lowest in the month of November (Figure 1).

**Figure 2:**
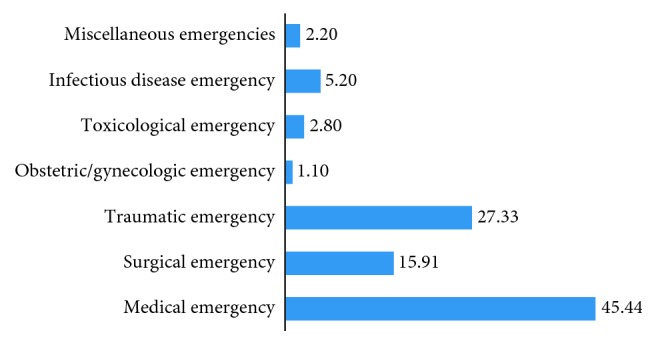
Major kinds of observed emergency conditions in the selected hospitals in Nekemte, western Ethiopia, 2017.

As to the spectrum and forms of adult ED admissions, 45.4% of the patients were of medical emergency in their types. Traumatic emergency (27.3%) and surgical emergency (15.9%) took the 2nd and 3rd ranks, respectively (Figure 2).

### 3.5. Nature of Emergency Department Admissions

**Figure 3:**
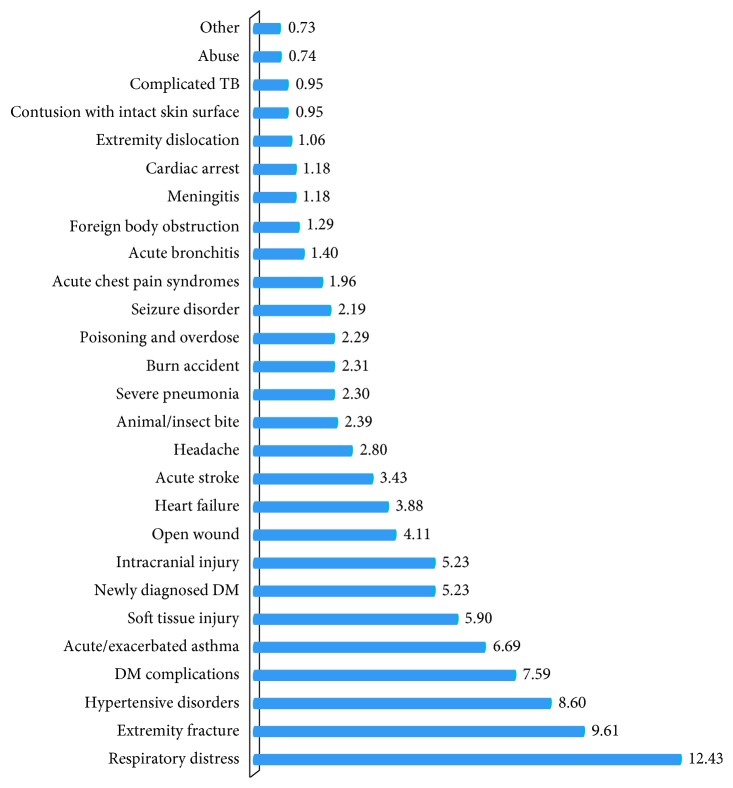
Leading causes of adult emergency department admission in the selected hospitals, Nekemte, Ethiopia, 2017.

Findings regarding the leading causes of adult emergency department admission at the selected hospitals revealed that a respiratory distress (12.43%), extremity fractures (9.61%), and hypertensive disorders (8.6%) were among the top leading causes of adult ED admissions (Figure 3).

Specific medical emergencies as leading causes of adult emergency room admissions were separately identified in this study. Shock of different causes, unconsciousness of different causes, respiratory distress, DM complications (DKA and hypoglycemia), nonspecified acute febrile illnesses, and serious forms of tuberculosis were among the top leading causes of ED admissions in the selected hospitals during this specified study period (Table 4).

Among the injuries presented, extremity fracture, organophosphate poisoning, and road traffic accident were among the leading causes of ED admissions in the selected hospitals. Impairment of breathing (37%) and circulatory compromises (30%), the two most common types of life-threatening problems, identified immediately on arrival (Table 5).

### 3.6. Clinical Outcomes and Duration of Hospital Stay

**Figure 4:**
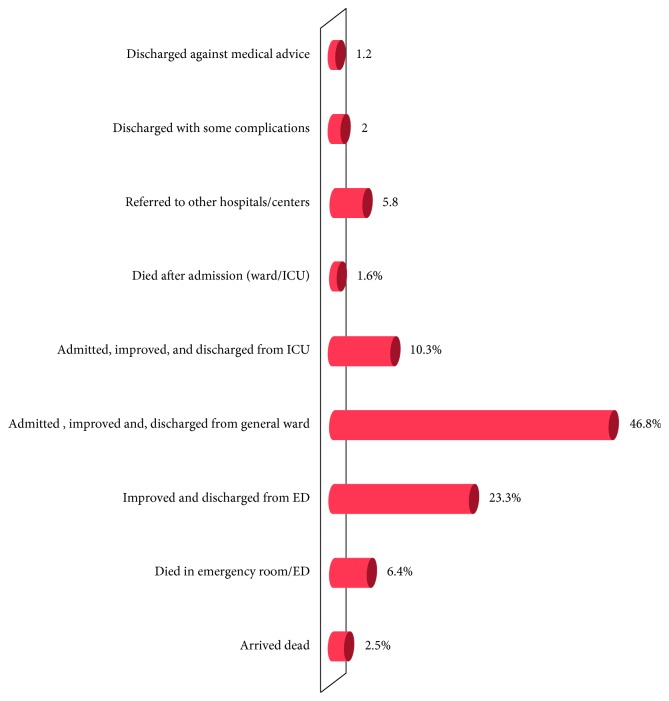
Distribution of respondents by their consequences of emergency department visits in Nekemte, western Ethiopia, 2017.

The final clinical outcome of emergency department admissions in terms of death, survival, and length of hospital stay was one aim of this study. While the large majority (90.9%) of them survived, the final outcome was not identified in about 1.5% of the patients. Overall, among all patients who visited emergency departments in the study period, about 8.5% (2.5% arrived dead, 6.4% died in the emergency departments, and the remaining 1.6% died after ward/ICU admission) (Figure 4).

Regarding the patients' duration of hospital stay, more than half (58.2%) cases stayed in respective hospitals for 1–3 days. The rest of 41.8% have stayed in the hospitals for 4–7 days.

## 4. Discussion

A number of studies revealed that there has been a consistent increase in the absolute number of emergency department admissions rate for defined population over the last decade. The key findings of this study related to pattern, characteristics, baseline clinical information, spectrum of emergency department admissions, leading causes of emergency department admission, and outcome of adult emergency department (ED) admissions of this study are compared and contrasted with currently existing literatures and presented briefly.

The mean age of the patients admitted to adult ED of the hospitals during the study period was 34.98 years. The adult ED admissions in this study were also found to be increasing progressively with age to reach peak at 15–24 years implying higher burden of diseases in the economically productive age group. Both findings are in line with similar studies conducted to determine the ED admission patterns in Nigeria [2, 5, 10, 12].

The decision to seek medical attention and the choice of healthcare provider are linked with the level of urgency of the complaint. Cases where there is little or no scope for choice refer to serious emergencies where immediate care is required [4, 11]. Also, the study explored respondents' relevant reasons of emergency room visits. While one-fourth (20.4%) of them arrived by ambulances (without identifying reason), only 40 (4.5%) of them visited the ED because they believe they get appropriate care at emergency rooms. About 23.6% of the patients visited the hospitals because they had no other place to go. A similar study done by Gindi et al. to assess emergency room utilization among adults aged 18–64 years found that about 79.7% of adults have visited the emergency room due to lack of access to other providers. Another similar study indicated that more than 66.0% who visited the ED was due to seriousness of the problems. One study, on the contrary, found that more than one-half of adults who have visited the emergency room was because of their perception that only the hospitals could provide help [3, 6, 9].

The admission patterns in the emergency room may reflect the actual burden of diseases in the immediate environment which also helps the health facilities to have a sound plan. In this study, the number of adult ED admissions per month was variable with peak admissions in the month of February and the lowest admission was in the month of November. We found that medical emergencies were found to be more frequent (45.4%) causes of adult ED admissions followed by traumatic emergencies (27.3%). The findings are in line similar other studies with little disparity from the study conducted at the University of Harcourt Teaching Hospital, in Nigeria which showed that only 17.3% (lower than 45.4% of this study) had medical emergencies [3, 6–11, 13, 14]. The difference could be indicative of the rising epidemics of noncommunicable diseases in the study area related with the lifestyle changes.

The outcome of patients admitted to adult ED was found to vary with patients' demographic and clinical characteristics. Overall, among patients who visited ED of the selected hospitals in this study, about 8.5% died (2.5% arrived dead, 6.4% died in ED, and the remaining 1.6% died after ward/ICU admission). Out of all patients presented to the selected hospital in the area, vast majority (90.9%) of them have survived after receiving treatment. The final outcome has not been identified in about 1.5% of patients due to referral and discharge against medical advice (DAMA). The ED mortality rate (8.5%) in this study is much greater than the finding of similar study conducted in Addis Ababa. The observed discrepancy might have been due to differences in the standard of emergency department care between the two areas. Similarly, the mean length of stay in hospitals is much longer than what has been reported in Addis Ababa and consistent with studies conducted in other similar settings [8, 15, 16].

This study has also revealed that impairment of breathing in about 37% followed by circulatory compromises in nearly 30% the emergency patients was observed as life-threatening problems. This is in line with the report found from UK, Nigeria, and similar other surveys but with slight variation with the study done in Ethiopia [9, 17, 18].

## 5. Conclusions

This study showed the selected hospitals received mixed cases of emergency conditions which also ended up with significant proportion of adult death. The admission rate was found to be varying with seasonal difference. The outcome of patients admitted to adult ED was found to vary with patients' demographic and clinical characteristics. Higher proportion of the cases required admissions to the wards/ICUs implying the seriousness of the encountered emergencies. A need for action to improve the readiness of existing emergency room services and investing further on standardizing and organizing prehospital services at the community level.

## Figures and Tables

**Table 1:** Distribution of respondents by their socio-demographic characteristics in Nekemte, western Ethiopia, 2017.

No.	Variables (*N* = 889)	Category	Frequency	Percent
1	Age of the patient	15–24	243	27.31
		25–34	215	24.20
		35–44	201	22.62
		45–54	127	14.30
		55–64	63	7.10
		+65	40	4.54

2	Sex	Male	503	56.60
		Female	386	43.41

3	Marital status	Single	273	30.70
		Married	542	61.02
		Divorced	46	5.20
		Widowed	28	3.10

4	Level of education attained	No schooling	257	28.92
		Primary (grade 1–6)	247	27.81
		Secondary (grade 7–12)	247	27.80
		Tertiary (>grade 12)	138	15.50

5	Monthly income	150–500	485	54.59
		500–750	117	13.20
		750–1500	54	6.13
		1500–3,000	83	9.30
		3000–5000	53	6.05
		+5000	55	6.20
		Unknown	42	4.70

6	Residence	Town	396	44.50
		Rural	493	55.50

7	Occupation	Government employee	111	12.50
		Maid/servant	34	3.80
		Merchant	133	15.07
		Farmer	351	39.50
		Laborer	30	3.40
		Driver	82	9.22
		No formal job	148	16.71

**Table 2:** Distribution of emergency patients by their baseline information, Nekemte, Ethiopia, 2017.

No.	Baseline information (*N* = 889)	Category	Frequency	Percent
1	Mode of arrival	By people/shoulder	65	7.30
		Public transportation	346	38.91
		Ambulance	282	31.72
		Private car	134	15.11
		On foot	59	6.48
		Horse back	3	0.30

2	Treatment given before arrival	Yes	266	29.81
		No	623	70.10

3	Source of referral	Self	461	51.90
		Public health center	304	34.22
		Other public hospitals	37	4.22
		Private health facility	38	4.30
		Other	49	5.51

4	Living condition	Alone	44	4.90
		With family	763	85.80
		With friends	51	5.70
		On the street	14	1.61
		Other	17	1.90

5	Number of visit	First	684	76.90
		Repeated	202	22.70
		Unknown	3	0.30

6	Arrival time	Weekend	96	10.81
		Night	135	15.10
		Day	658	74.10

7	Time between arrival and initiation of treatment in minutes	<15	662	74.50
		15–30	166	18.71
		30–60	48	5.42
		>60	13	1.50

8	Who brought the patient	Self	475	53.40
		Friend	300	33.70
		Family member	109	12.32
		Police	5	0.60

9	Vital signs on arrival	Normal	348	39.11
		Deranged	528	59.42
		Not taken	13	1.51

10	Level of consciousness on arrival	Conscious	404	45.41
		Subconscious	268	30.14
		Unconscious	127	14.33
		Not evaluated	90	10.22

11	Presence of known chronic illness	Yes	259	29.10
		No	591	66.55
		Unknown	39	4.40

12	Distance of patient's home and hospital in kilometers	<5	350	39.41
		5–15	242	27.22
		16–25	79	8.93
		26–35	51	5.71
		36–45	44	4.90
		>45	123	13.86

**Table 3:** Respondents' main reason for emergency room visit in Nekemte, western Ethiopia, 2017.

No.	Main reason for emergency room visit	Frequency	Percent
1	Health provider advised to go	233	26.20
2	The problem was so serious that other clinics cannot help	81	9.11
3	Arrived by ambulance or other emergency vehicles	181	20.38
4	The patient had no other place to go	207	23.30
5	Emergency room is the closest provider	85	9.61
6	Get most of care at the emergency room	40	4.50
7	Unidentified reason	62	7.04
Total		889	100.0

**Table 4:** Identified medical emergencies as leading causes of emergency room admission, Nekemte, Ethiopia, 2017.

Forms of medical emergencies	Frequency	Percent
*Cardiovascular (N* *=* *257)*		
Acute chest pain syndromes	12	4.63
Hypertensive disorders	71	27.62
Heart failure	29	11.22
Shock	140	54.41
Cardiac arrest	5	1.90

*Neurology (N* *=* *172)*		
Meningitis	5	2.90
Stroke	25	14.50
Seizure disorder	14	8.11
Headache	11	6.31
Unconsciousness (coma)	117	68.00

*Respiratory (N* *=* *192)*		
Respiratory distress	105	54.60
Foreign body obstruction	3	1.55
Asthma	54	28.11
COPD	5	2.63
Severe pneumonia	15	7.86
Bronchitis	7	3.61
Complicated TB	3	1.54

*Endocrine (N* *=* *96)*		
Diabetes mellitus	34	35.43
DM complications	62	64.50

**Table 5:** Emergency room admission due to traumatic and toxicologic emergencies, in Nekemte, western Ethiopia, 2017.

	Frequency	Percent
*Forms of traumatic injury (N* *=* *483)*		
Contusion with intact skin surface	3	0.65
Soft tissue injury	47	9.71
Intracranial injury	41	8.40
Fracture	80	16.50
Dislocation	4	0.81
Foreign body obstruction	6	1.22
Burn	69	14.22
Falling accident	37	7.61
Road traffic accident	99	20.41
Machinery injury	6	1.22
Fighting	72	14.90
Other physical abuses	14	2.82
Sexual abuse	5	1.00

*Toxicology (N = 33)*		
Suicide	12	22.20
Human bites	3	5.50
Animal bites	10	18.51
Insect bites	3	5.52
Organophosphate poisoning	18	33.30
Carbon monoxide poisoning	2	3.72
Drug overdose	3	5.51
Unidentified	3	5.49

## Data Availability

The data used to support the findings of this study are available from the corresponding author upon request.

## Abbreviations

AGE: Acute gastroenteritis
CCU: Critical care unit
CHF: Congestive heart failure
DAMA: Discharge against medical advice
DKA: Diabetic ketoacidosis
ED: Emergency department
ENT: Ear nose throat
ICU: Intensive care unit
NCD: Noncommunicable disease
RTA: Road traffic accident
SSA: Sub-Saharan Africa
UK: United Kingdom.

## Additional Points

*Limitation*. As this was an institution-based study, the research did not know the fate of patients who have not visited the selected health facilities. In addition, the study did not identify the final outcomes of patients who were either referred to other facilities or discharged against medical advice.
